# AN EXPLORATION OF HOME CARE NURSES’ EXPERIENCES IN DEPRESCRIBING OF MEDICATIONS FOR OLDER ADULTS IN THE COMMUNITY

**DOI:** 10.1093/geroni/igz038.2607

**Published:** 2019-11-08

**Authors:** Winnie Sun

**Affiliations:** University of Ontario Institute of Technology, Oshawa, Ontario, Canada

## Abstract

The aim of this study is to explore the barriers and enablers of deprescribing from the perspectives of home care nurses, as well as to conduct a scalability assessment of an educational plan to address the learning needs of home care nurses about deprescribing. This study employed an exploratory qualitative descriptive research design, using scalability assessment from two focus groups with a total of eleven home care nurses in Ontario, Canada. Thematic analysis was used to derive themes about home care nurse’s perspectives about barriers and enablers of deprescribing, as well as learning needs in relation to deprescribing approaches. Home care nurse’s identified challenges for managing polypharmacy in older adults in home care settings, including a lack of open communication and inconsistent medication reconciliation practices. Additionally, inadequate partnership and ineffective collaboration between inter-professional healthcare providers were identified as major barriers to safe deprescribing. Further, home care nurses highlighted the importance of raising awareness about deprescribing in the community, and they emphasized the need for a consistent and standardized approach in educating healthcare providers, informal caregivers, and older adults about the best practices of safe deprescribing. Nurses in home care play a vital role in medication management and, therefore, educational programs must be developed to support their awareness and understanding of deprescribing. Study findings highlighted the need for the future improvement of existing programs about safer medication management through the development of a supportive and collaborative relationship among the home care team, frail older adults and their informal caregivers.

